# The Presence of Precursors of Benign Pre-B Lymphoblasts (Hematogones) in the Bone Marrow of a Paediatric Patient with Cytomegalovirus Infection

**DOI:** 10.4137/cmo.s751

**Published:** 2008-05-20

**Authors:** F Moreno-Madrid, J Uberos, M Díaz-Molina, A Ramírez-Arredondo, P Jiménez-Gámiz, A Molina-Carballo

**Affiliations:** 1 Servicio de Pediatría; Ciudad Sanitaria Virgen de las Nieves. Granada (Spain); 2 Distrito Sanitario Granada. Granada (Spain); 3 Servicio de Hematología; Ciudad Sanitaria Virgen de las Nieves. Granada (Spain)

**Keywords:** hematogones, pre-B cells, cytomegalovirus, infants, leukemia, acute lymphoblastic leukemia

## Abstract

Hematogones are normal B-lymphoid precursors that multiply in the bone marrow of small children and of adults with ferropenic anaemia, neuroblastoma or idiopathic thrombocytopenic purpura. They are not normally found in peripheral blood, and the immunophenotype is virtually indistinguishable from that of B lymphoblasts. We discuss the case of a 3-month infant with an active cytomegalovirus infection, with hepatitis and pancytopenia associated with 13% hematogones in the bone marrow.

## Introduction

Hematogones are B-lymphocyte precursors that are made apparent by the existence of a uniform nuclear chromatin, by the scarce presence of cellular cytoplasm in bone marrow extension, by the coexpression of CD-10, CD-38 and CD-19, and by the non expression of surface immunoglobulin. They are found in small concentrations in healthy people, but their numbers may increase among children or adults undergoing various pathological processes, with lymphomas, non-neoplastic blood cytopenias, post-chemotherapy, post-bone marrow transplant and HIV being the most common such situations [1, 2]. In some patients, hematogones may represent over 50% of bone marrow cellularity; in such cases, these cells can give rise to problems of differential diagnosis with acute lymphoblastic leukemia or lymphoblastic lymphoma, due to the considerable similarity in their cell morphology. Occasional reports have been made of the presence of hematogones in patients with myeloblastic leukemia or Down syndrome [3]. Hematogones have also been identified in the cells of the bone marrow of premature neonates several weeks after birth, with a presence of up to 25% at two weeks of life [4]. Exceptionally, hematogones have been observed in the bone marrow extension of patients with cytomegalovirus infection or thrombocytopenia [5]. Although there have been reports of an increase in the percentage of hematogones in patients with immune-mediated thrombocytopenia, there does not seem to be a proven relation between the intensity of the thrombopenia, as assessed by a count of platelets in the peripheral blood, and the percentage of hematogones in the bone marrow [2].

We describe the case of a 3-month infant with active cytomegalovirus infection with hepatitis and pancytopenia, associated with a presence of 13% hematogones in the bone marrow.

## Case Report

Infant aged 3 months, born prematurely (at 35 weeks’ gestational age) with a weight at birth of 3100 g, admitted to the Neonatal Unit at our hospital. Discharged after one week, in view of the absence of nutritional, respiratory or metabolic disorders. Brought to the Emergency Service at our hospital after suffering high temperatures over three days. Physical examination revealed only an unusually pale skin and mucosal surfaces, together with a hepatosplenomegaly of 3 cm beneath the costal margin; the abdomen was soft and not tender to palpation. No wheezing was heard and the heart tone was normal.

### Peripheral blood analysis

47% mature lymphocytes, 2% NK cells, 14% B lymphocytes, absence of B-precursor cells with CD20/CD10 expression. The remaining cells were composed of mature granulocyte. Leucocytes 4.3 × 10^3^/μl, Hemoglobin 8 g/dl, Haematocrit 26.6%, Platelets 119 × 10^3^/μl, Glucose 4.8 mmol/L, Creatinine 0.027 mmol/L, Urea 1.7 mmol/L, Uric acid 0.030 mmol/L, Total cholesterol 3.02 mmol/L, Triglycerides 2.56 mmol/L, AST 520 U/L, ALT 403 U/L, Gammaglutamyl-transpeptidase 262 U/L, Alkaline phosphatase 209 U/L, Total bilirubin 0.021 mmol/L, Calcium 2.37 mmol/L, Phosphorus 1.58 mmol/L, Iron 0.006 mmol/L, Sodium 140 mmol/L, Potassium 5.1 mmol/L, LDH 1083 U/L.

### Bone marrow analysis

5% blasts (myeloids and lymphoids), 7% mature lymphocytes (3% CD4 T cells, 3% T CD8 cells, 1% NK cells), 1% B lymphocytes, 15% pre-B cells with CD20/CD10 expression, 2% monocytes. Negative expression of CD-44 and CD-54. The remaining cells were mainly comprised of the mature granulocytic myeloid series.

Hemoculture, uroculture, coproculture and cerebrospinal fluid culture: negative.

IgM serology to cytomegalovirus: positive. Cytomegalovirus antigen in urine: positive.

HIV, Epstein-Barr, Toxoplasma, Parvovirus B19, Lues disease, Mycoplasma pneumoniae, Brucella, Chlamidia, Hepatitis B and A, Rubeola, Leishmania: negative.

Mantoux: negative. Eye background: normal. Abdominal radiography confirmed the existence of hepatosplenomegaly and the absence of free peritoneal liquid. Chest radiology showed normal pulmonary parenchma, with no appearance of cardiomegaly.

The patient’s temperature returned to normal after 12 days; the patient remained free of fever for 6 days, after which it returned; blood and urine cultures were negative. The count for the white, red blood cells and megakaryocytic series remained unchanged. The patient was treated initially with ganciclovir at a dose of 25 mg/12 h. Hematologic and biochemical parameters subsequently returned to normal, and the patient remained clinically asymptomatic.

## Discussion

Hematogones are normal B-lymphoid precursors that multiply in the bone marrow of small children and of adults with ferropenic anaemia, neuroblastoma or idiopathic thrombocytopenic purpura. They also occur frequently in regenerative bone marrow after chemotherapy or bone marrow transplant, but are not normally found in peripheral blood. The immunophenotype is virtually indistinguishable from that of B lymphoblasts. Thus, these cells express markers associated with lymphoblasts, such as TdT, CD10 and CD34. What differentiates them from the latter is the continuity of the antigen expression, which means that hematogones are undergoing a process of maturation, and so it is possible to detect the gradual expression of certain antigens, for example CD20 and surface immunoglobulins.

The presence of hematogones may create some confusion in flow cytometry analysis with the cell-count of acute lymphoblastic leukemia in the bone marrow, given the similarity of its immunophenotype to that of pre-B lymphoblasts. Various authors have reported the positivity of hematogones to CD-10, CD-19, CD-20 and HLA-DR [5], and also that hematogones may occasionally express TdT and CD-34. The negativity to CD-44 and CD-54 has been used to discriminate between hematogones and leukemic blasts [6]. In the present case, we observed the expression of 15% CD-20/CD-10 lymphoblasts in the bone marrow.

The presence of hematogones has been reported among children during the process of cellular regeneration of the bone marrow, following certain viral infections [6]; however, there are very few references in the literature to the presence of hematogones in the bone marrow following cytomegalovirus infection [5].

We conclude that during infancy the diagnosis of acute lymphoblastic leukemia of the pre-B cells should be explicitly distinguished from the benign proliferative states of pre-B cells (hematogones), especially after infection by cytomegalovirus.

## Figures and Tables

**Figure 1:**
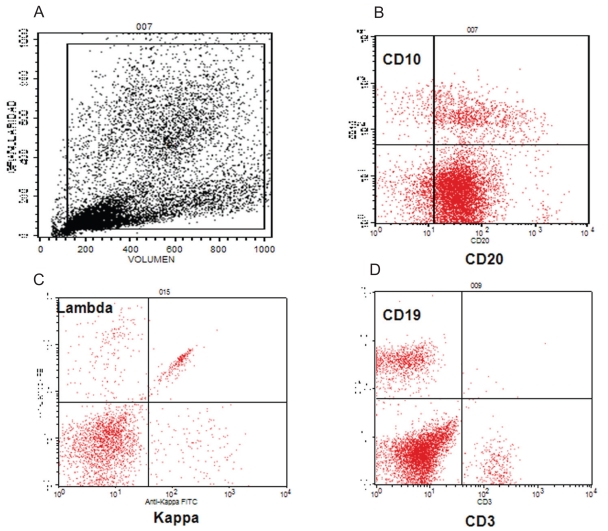
Analysis of the bone marrow by flow cytometry. Note the positive expression of the CD19 marker of the B lymphocytes (**D**), the gradual expression of CD20 (**B**) and the expression of surface immunoglobulins. Also detected were cells with a positive expression with a polyclonal pattern (the expression of kappa and lambda light chains) and cells that do not express IgS (**C**).
